# 

**Published:** 1846-09

**Authors:** 


					Vol. VII.
SEPTEMBER, 1846.
? % ? . ri * ?3 ' iv,
wm
r
or
A '
r';%-4 + $? .f t .3. V-
j* *> --??'"' ?' - - /rjtfS', ^
PfP
58
I 8 H E D
QUARTER LY.
JitBtiaHBIX- UNDER THE AUSPICES OF
.7i fe
tan *.(!??S ? ?<?"? auC
&g?jS?55s# Si' -
M. D? D. D
AMOS WBSTCOTT, M. D.^X). D. S.
gf&
Tmmi
JOHN W. WOODS, POINTER.
*
? 5 0O per annum, payable in advance,

				

